# Human Expertise Outperforms Artificial Intelligence in Medical Education Assessments: MCQ Creation Highlights the Irreplaceable Role of Teachers

**DOI:** 10.1055/s-0045-1813040

**Published:** 2025-11-19

**Authors:** Aliya Mufti, Kashif Ali, Gaurav Sharma, Mohammad Saleem

**Affiliations:** 1 Department of Physiology, All India Institute of Medical Sciences, New Delhi, National Capital Territory of Delhi, India; 2 Department of Physiology, Jamia Millia Islamia, New Delhi, New Delhi, National Capital Territory of Delhi, India; 3 Department of Physiology, All India Institute of Medical Sciences, Rajkot, Gujarat, India; 4 Department of Pathology, Jamia Millia Islamia, South East, Okhla, National Capital Territory of Delhi, India

**Keywords:** artificial intelligence, medical education, multiple-choice questions

## Abstract

**Introduction:**

Multiple-choice questions (MCQs) are vital tools for assessment in education because they allow for the direct measurement of various knowledge, skills, and competencies across a wide range of disciplines. While artificial intelligence (AI) holds promise as a supplementary tool in medical education, particularly for generating large volumes of practice questions, it cannot yet replace the nuanced and expert-driven process of question creation that human educators provide. This study seeks to close the gap, particularly with regard to difficulty index, discrimination index, and distractor efficiency.

**Materials and Methods:**

A total of 50 medical students received a set of fifty randomized, blinded, validated MCQs by human physiology experts. Of these, 25 were made by AI, and the remaining 25 were made by qualified, experienced professors. Using the item response theory (IRT) framework, we calculated key metrics like item reliability, difficulty index, discrimination index, and distractor functionality.

**Results:**

The results demonstrated that the difficulty index of AI-generated MCQs (mean = 0.62, SD = 0.14) was comparable to that of expert-generated questions, with no statistically significant difference observed ( *p* = 0.45). However, significant differences emerged in other key quality metrics. The discrimination index, which reflects a question's ability to distinguish between high- and low-performing students, was notably higher for expert-created MCQs (Mean = 0.48, SD = 0.12) than for those generated by AI (Mean = 0.32, SD = 0.10), indicating a moderate-to-large effect (p = 0.0082, Chi-square = 11.7, df = 3). Similarly, distractor efficiency (DE), which evaluates the effectiveness of incorrect answer options, was significantly greater in expert-authored questions (Mean = 0.24, SD = 7.2) compared to AI-generated items (Mean = 0.4, SD = 8.1), with a moderate effect size (p = 0.0001, Chi-square = 26.2, df = 2). These findings suggest that while AI can replicate human-level difficulty, expert involvement remains crucial for ensuring high-quality discrimination and distractor performance in MCQ design.

**Conclusion:**

The findings suggest that AI holds promise, particularly in generating questions of appropriate difficulty, but human expertise remains essential in crafting high-quality assessments that effectively differentiate between levels of student performance and challenge students' critical thinking. As AI technology continues to evolve, ongoing research and careful implementation will be essential in ensuring that AI contributes positively to medical education.

## Introduction

Artificial intelligence (AI) was invented in 1956 by pioneers like John McCarthy, Marvin Minsky, Nathaniel Rochester, and Claude Shannon. 1 The first AI program was known as the logical theorist, which was developed by Newell and Simon. This program could mimic a few aspects of human problem-solving abilities. 2 Early developments in AI include the Turing Test in 1951, which measured the machine's ability to exhibit intelligent behavior that could be mistaken for human. 3 Similarly, the first Chatbot, ELIZA, was created by Joseph Weizenbaum in 1959 and demonstrated the ability to simulate conversation. 4 By 1965, programs like MYCIN and DENDRAL were making progress in the fields of healthcare and chemistry, treating blood infections and aiding in chemical analysis, respectively. 5 With time, AI has expanded its influence to almost every sector, including banking, healthcare, finance, agriculture, transport, and, notably, education. 6

AI is often viewed as one of the most groundbreaking technologies in human history. In the field of education, AI has the capability to revolutionize learning by making it an experience tailored for individual students, thus making education more engaging and efficient. Tools such as intelligent tutoring systems, chatbots, and automated grading systems are not only making educational processes more efficient but also helping teachers by saving time and providing consistent feedback. 7

In the last decade, a significant rise has been seen in the use of AI in higher education. AI tools have become more accessible and affordable for both educators and students, thus making intelligent tutoring, learning management, and even predicting student performance. 8 AI assistants like Google Gemini, ChatGPT, Metaverse Llama, and Microsoft Copilot are increasingly being used in educational settings, helping both teachers and students.

The impact of AI is also being felt in the field of medical education. AI has been utilized to develop, analyze, and refine medical curricula, as well as to enhance learning and assessment processes. 9 Assessment is a cornerstone of the educational system and plays a critical role in documenting the knowledge, skills, attitudes, and beliefs of students. 10 Assessment greatly influences the teaching and learning process across all levels of education. The method of assessment employed defines the knowledge and skills students acquire and how they apply them in their future. Additionally, assessment helps institutions measure the success of their courses or curriculum by evaluating students' performances. 11

In recent years, medical education has shifted from a teacher-centered to a student-centered approach. Didactic lectures, which are often passive and yield poor outcomes, are being replaced by innovative teaching methods to improve learning outcomes. Many medical schools are now incorporating various pedagogical strategies, such as interactive question-and-answer sessions, case-based learning, assignments, and regular tests, to make learning more effective and participatory. 12 Students generally perceive regular tests as beneficial tools for reinforcing their learning, a phenomenon known as the testing effect. 13 Beyond being a tool for assessment, tests are also powerful learning aids. Among various testing methods, multiple-choice questions (MCQs) remain a popular choice in education. MCQs are objective and have a predetermined correct answer, making them a reliable assessment tool with bias.

MCQs are widely accepted in medical education for several reasons. They save time by allowing for quick and easy evaluation, help teachers identify learning difficulties, and provide rapid feedback to students. Additionally, MCQs are enjoyable, highly motivating, and help in building students' confidence. 14 Evidence suggests that MCQs enhance learning, improve recollection and reflection on discussed topics, and lead to better performance in summative assessments. 15 MCQs can also assess higher-order cognitive skills such as interpretation, application, and evaluation, in addition to knowledge recall. They are reproducible, reliable, and cost-effective. 16

However, despite their advantages, creating well-constructed MCQs is a time-consuming process that requires formal training. AI has the potential to make the assessment process more accurate, rapid, and cost-effective, while also providing detailed and customized feedback. Previous studies have explored the use of AI in generating MCQs. For example, a study in 2022 demonstrated the capability of a program to generate MCQs from raw text. 17 Another study in 2024 highlighted the potential of ChatGPT as a tool for test development. 18 Although one study found that ChatGPT 3.5 did not consistently produce high-quality MCQs, it suggested that teachers could still benefit from using AI-generated questions as a starting point, requiring only minor adjustments rather than creating new questions from scratch. 19

The integration of AI into education, especially in creating assessment tools, is rapidly gaining interest. AI-generated MCQs offer benefits such as scalability, efficiency, and cost-effectiveness. However, there is still limited research comparing the quality and effectiveness of AI-generated MCQs with those created by human experts. This study aims to bridge that gap by systematically comparing AI-generated MCQs with expert-generated MCQs in the context of medical education. Our research aims to determine whether AI-generated MCQs can match or exceed the quality of human expert-generated MCQs in medical education assessments, specifically in terms of difficulty index, discrimination index, and DE.

## Materials and Methods

**Study design:** This study employed a cross-sectional study design and was conducted in a medical college in India after taking approval from the Institutional Ethical Committee. A total of 50 medical students were given a set of 50 randomized and blinded MCQs, comprising 25 MCQs by trained and experienced teachers and 25 MCQs generated by AI.

**Fig. 1:**
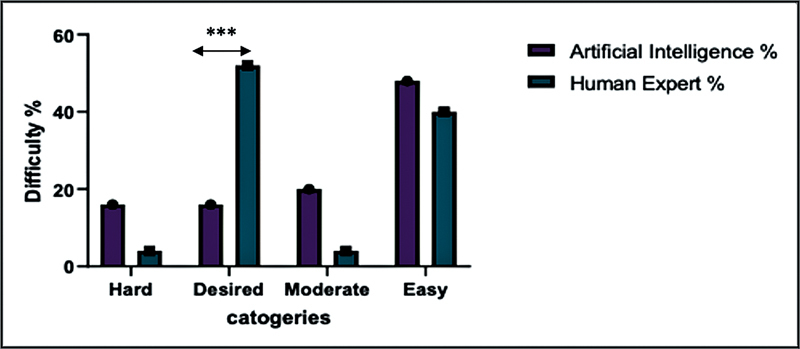
Difficulty index comparing artificial intelligence and human experts across four categories: hard, desired, moderate, and easy. The hard category represents tasks that pose significant challenges to both AI and human experts. The desired zone indicates the optimal level of difficulty where both AI and humans perform efficiently, making it the most suitable range for assessment. The moderate category includes tasks of average difficulty that require a reasonable level of effort and expertise. Lastly, the easy category encompasses tasks that are simple and straightforward for both AI systems and human experts.

**Fig. 2:**
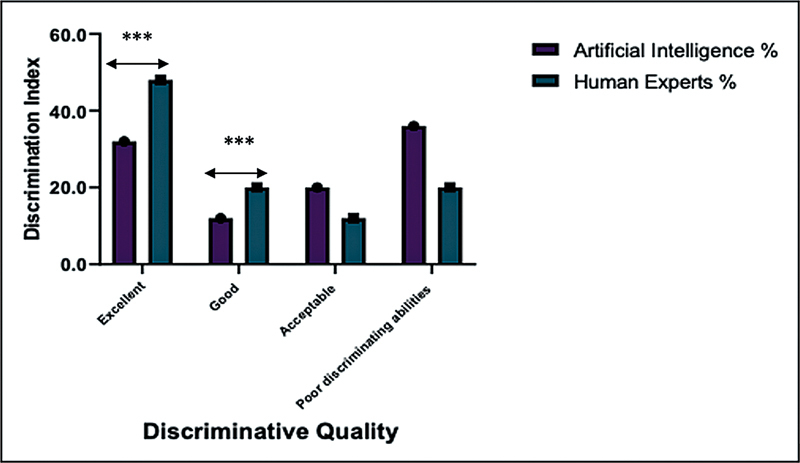
The distractor analysis based on the discrimination index, comparing the performance of artificial intelligence and human experts across four categories: excellent, good, acceptable, and poor. The excellent category reflects items that strongly differentiate between high and low performers, indicating high-quality questions. The good and acceptable categories represent questions with reasonable discriminating power, still useful for assessment purposes. In contrast, the poor category includes items with limited or no ability to distinguish between different levels of performance, suggesting the need for revision. This analysis helps evaluate the effectiveness of each distractor in assessing knowledge accurately.

**Process of human expert-generated MCQs:** We requested 30 teachers of physiology teaching in various colleges across India to participate in the study, and after explaining it to them, 24 teachers with the minimum required teaching experience of > 5 years promptly responded. They were asked to prepare MCQs with the instructions given in Fig. 1 . After collecting the MCQs from each participating teacher, 506 questions on a total of 10 themes were available. Randomization was used to collect 50 questions from 10 themes ( Fig. 2 and Supplementary material (available in the online version only)).

**Selection of AI tools:** We employed four leading AI tools for generating MCQs: Google Gemini, Metaverse Llama, Microsoft Copilot, and ChatGPT. These tools were chosen due to their advanced natural language processing capabilities, which are well-suited for generating educational content. The MCQs were created by inputting the prompt given in Fig. 1 . A total of 386 MCQs were created by the four AI tools, and a random selection process was used to shortlist 50 AI-generated MCQs set from the total of 386 MCQs.

**Validation process:** A panel of five subject experts who were not part of the study was assigned for validation of the two sets of MCQs after blinding for the origin (human/AI) of the questions. The validation criteria included relevance to the curriculum, clarity of the questions, cognitive level, and the appropriateness of distractors. This process ensured that both sets of MCQs were suitable for assessing the intended learning outcomes. Three questions from a set of 50 MCQs prepared by human experts and 7 questions from a set of AI-prepared 50 MCQs were excluded due to various reasons (relevance to the curriculum, clarity of the questions, cognitive level, and the appropriateness of distractors). A final question paper consisting of 50 MCQs was prepared by randomly selecting 25 questions each from human experts and AI question sets, which originally contained 47 and 43 MCQs, respectively. Students were instructed to fill in their personal details and provide feedback ( Fig. 2 ).

### Study Participants

Medical students were notified of a fixed date, time, and venue of the test one month prior to the test. The test could be taken by any student who had completed the final year of MBBS in the last 5 years. There was no negative marking, a maximum of 200 marks for 50 MCQs, and 4 marks were awarded for each correct answer. Students were encouraged to complete the exam on time. After completing the test, the students' responses were evaluated manually, and questions were regrouped back to AI and human experts ( Fig. 2 ).

**Fig. 3:**
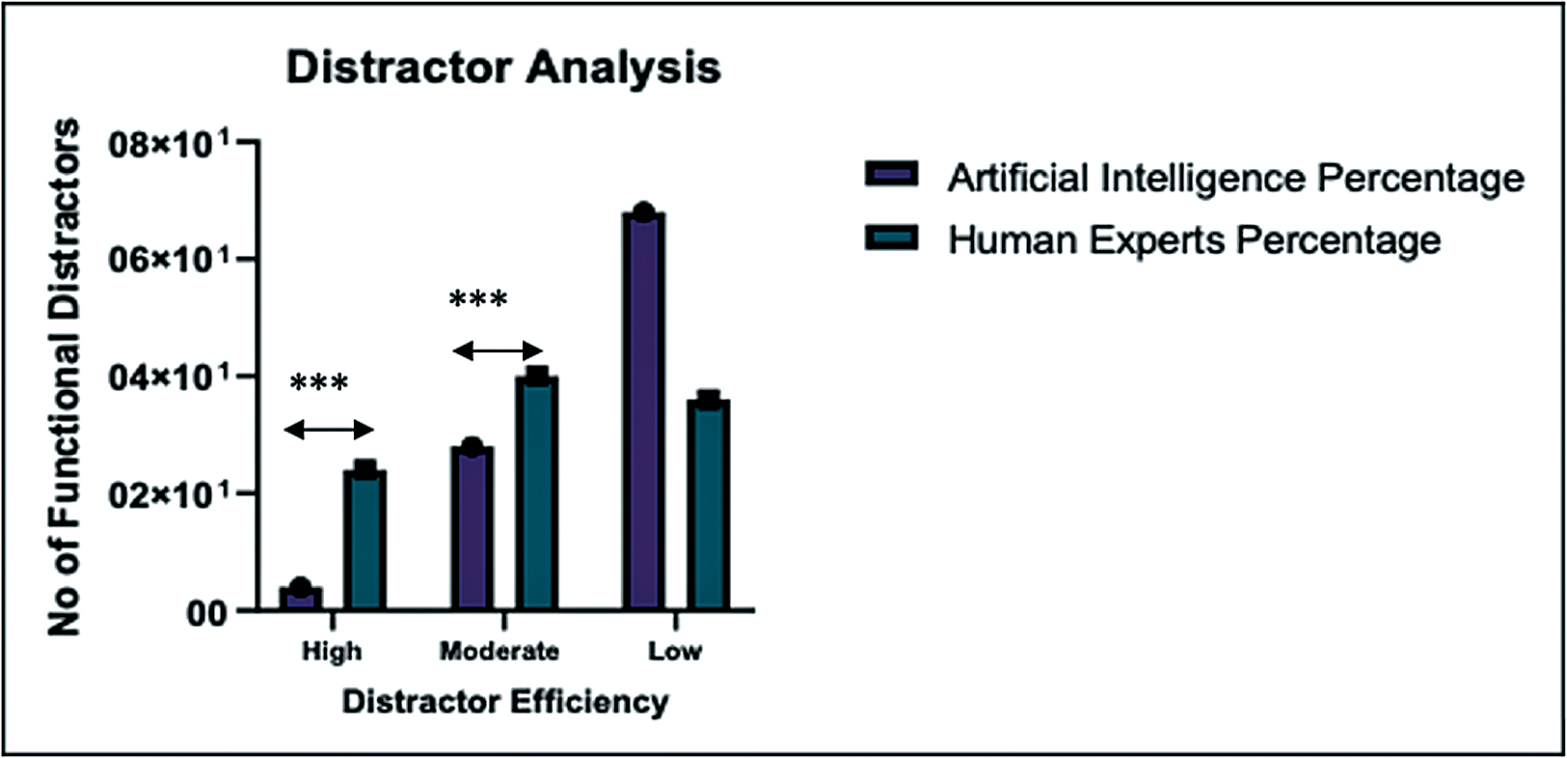
Functional distractors: number of functional distractors per Item. This figure categorizes the number of functional distractors used in multiple-choice questions, comparing the performance of artificial intelligence and human experts. Items are divided into three levels based on the number of functional distractors: 3, indicating high distractor efficiency, where all options were effective; 2, representing moderate efficiency, with most distractors being plausible; and 0–1, signifying low distractor efficiency, where few or no distractors successfully differentiated knowledge levels among test-takers. This analysis provides insight into the overall quality and effectiveness of distractor design in assessment items.

### Data Analysis

Using the item response theory (IRT) framework, we calculated key metrics like the item reliability, difficulty index, discrimination index, and distractor functionality.

We ascertained the proportion of MCQs that were categorized as difficult, desired, moderately easy, and easy in terms of difficulty level, and the question distribution using discriminating indices that were excellent, good, acceptable, poor, and negative. Furthermore, we also determined the percentage of MCQs that fell into categories of high, moderate, and low DE.

## Results

Of the 48 students who volunteered for the test, 42 completed it within the allotted time. The remaining six students were excluded from the results as they left the exam before the minimum duration of 10 minutes had passed. This was done to ensure the results were reliable and not based on incomplete data. The study focused on 42 medical students who were preparing for postgraduate entrance exams. These students, comprising 24 males and 18 females, took a physiology exam consisting of 25 MCQs created by AI and 25 MCQs created by human experts with at least 5 years of teaching experience in physiology ( Table 1 ). The analysis revealed no significant difference in performance between male and female students. The Kuder–Richardson Formula-20 score for the two sets of 25 MCQs prepared by human experts and AI was found to be 0.67 and 0.57, respectively.

**Table 1:** Descriptive data: participating teachers vs. participating students

Variable	Gender (M/F)	*N* (%), Participating teachers ( *n* = 24)	*N* (%), Participating students ( *n* = 42)
Gender	Male	14 (58.3%)	24 (57.1%)
	Female	10 (41.7%)	18 (42.9%)
Experience	5–10 y/student experience 0–1 y since MBBS	12 (50.0%)	30 (71.4%)
	10–20 y/student experience 2–3 y since MBBS	8 (33.3%)	11 (26.2%)
	>20 y/student experience 3–5 y since MBBS	4 (16.7%)	1 (2.4%)
Assessment results	Total MCQs	50	50
	Marks per MCQ	4	4
	Maximum marks	200	200
	Average percentage	70%	65%

## Difficulty Index Analysis

In our analysis of question difficulty, we found that AI-generated questions tended to be more challenging than those created by human experts. Specifically, 16% of the AI questions were classified as hard, while only 4% of the human experts' questions fell into this category ( Table 2 ). This indicates that AI may produce questions that are harder than those designed by humans.

**Table 2:** Difficulty index: artificial intelligence vs. human experts

Difficulty quality	Difficulty range (%)	Artificial intelligence, *N* (%)	Human experts, *N* (%)
Hard	0–29	4 (16%)	1 (4%)
Desired	30–70	4 (16%)	13 (52%)
Moderate easy	71–79	5 (20%)	1 (4%)
Easy	≥ 80	12 (48%)	10 (40%)

Statistical results: *χ* ^2^ = 37.6, df = 3, *p* = 0.0001.

When it comes to questions that fit within the desired difficulty range, human experts had a much higher percentage—52% of their questions were in this ideal range. In contrast, only 16% of the AI-generated questions were of the desired difficulty level ( Table 2 ). This suggests that human experts are better at creating questions that match the expected difficulty for medical students.

Additionally, AI-generated questions were more likely to be in the moderate-easy category, with 20% of the questions falling into this range, compared to just 4% of the human experts' questions ( Table 2 ).

Lastly, AI produced a higher percentage of easy questions (48%) compared to human experts (40%; Table 2 ). This shows that AI tends to generate questions that are easier, which might not challenge students as effectively as the questions created by human experts.

**Interpretation:** There is a highly significant difference between AI and human experts in difficulty level distribution. Human experts excel in producing Desired difficulty questions (52%), aligning well with educational assessment goals. AI, on the other hand, produces mostly easy questions (48%) and fewer desired ones (16%), indicating less optimal control over difficulty calibration.

**Conclusion:** Human experts show superior judgment in designing balanced, educationally appropriate questions. AI-generated items skew toward extremes (too easy or too hard), suggesting the need for better difficulty modeling.

## Discrimination Index Analysis

**Excellent discrimination:** Human experts created a higher percentage (48%) of questions that effectively distinguished between different levels of student ability, compared to AI-generated questions (32%). This suggests that questions created by human experts are better at evaluating varying levels of student performance ( Table 3 ).

**Table 3:** Discriminative quality of AI and human experts

Discriminative quality	Discrimination index	Artificial intelligence, *N* (%)	Human experts, *N* (%)
Excellent	DI > 0.4	8 (32%)	12 (48%)
Good	DI = 0.3–0.39	3 (12%)	5 (20%)
Acceptable	DI = 0.2–0.29	5 (20%)	3 (12%)
Poor discriminating abilities	DI < 0.2	9 (36%)	5 (20%)

Statistical results: Discrimination index, *χ* ^2^ = 11.7, df = 3, *p* = 0.0082.

**Good discrimination:** AI-generated questions had a lower percentage (12%) with good discrimination compared to those created by human experts (20%). This indicates that AI questions were less effective in differentiating between students' levels of understanding ( Table 3 ).

**Acceptable discrimination:** On the other hand, AI had a higher percentage (20%) of questions with acceptable discrimination than human experts (12%). This shows that while AI questions were sometimes adequate, they were less effective overall ( Table 3 ).

**Poor discriminating abilities:** AI also had a higher percentage (36%) of questions with poor discriminating abilities compared to human experts (20%). This means that many AI-generated questions did not effectively distinguish between students with different levels of ability ( Table 3 ).

**Interpretation:** There is a statistically significant difference between AI and human experts in discriminating ability distribution ( *χ* ^2^ = 10.78, *p* < 0.05). Human experts produced significantly more “excellent” and “good” items. AI generated more “poor” and “acceptable” items, indicating weaker discriminatory power.

**Conclusion:** Human experts produced more excellent and good questions (68% combined). AI generated more poor and acceptable ones (56% combined), indicating human experts have stronger discriminative abilities in question design.

## Distractor Analysis

When analyzing the efficiency of distractors, we found notable differences between MCQs generated by human experts and those created by AI ( Fig. 3 ).

**High distractor efficiency:** Human experts produced a higher percentage (24%) of questions with highly effective distractors compared to AI, which only had 4% of such questions ( Table 4 ). This suggests that human-generated questions are better at using distractors to challenge students.

**Table 4:** Functional distractors of AI and human experts

No. of functional distractors	Distractor efficiency	Artificial intelligence, *N* (%)	Human experts, *N* (%)
3	High	1 (4%)	6 (24%)
2	Moderate	7 (28%)	10 (40%)
0–1	Low	17 (68%)	9 (36%)

Statistical results: *χ* ^2^ = 26.2, df = 2, *p* = 0.0001.

**Moderate distractor efficiency:** Human experts also had a higher percentage (40%) of questions with moderately effective distractors, while AI produced 28% of such questions ( Table 4 ). This indicates that human experts are better at creating questions with distractors that are somewhat effective in assessing student understanding.

**Low distractor efficiency:** A significant difference was observed in questions with low DE. AI had a higher percentage (68%) of questions with distractors that were less effective, compared to human experts, who had only 36% ( Table 4 ). This shows that the distractors in AI-generated questions are generally less effective at differentiating between varying levels of student knowledge.

**Interpretation:** There is a statistically significant difference between AI and human experts in DE distribution. Human experts outperform AI, producing significantly more high- and moderate-efficiency distractors. AI tends to create a greater proportion of low-efficiency distractors, indicating less discriminative or less functional options.

**Conclusion:** AI produced a much higher percentage of low-efficiency distractors (68%), indicating weaker quality distractors. Human experts generated more high-efficiency (24%) and moderate-efficiency (40%) distractors, showing better balance and quality.

## Discussion

MCQs are vital tools for assessment in education because they allow for the direct measurement of various knowledge, skills, and competencies across a wide range of disciplines. They can test concepts, principles, judgment, inference, reasoning, data interpretation, and the application of information. 1 20 21 MCQs are also efficient to administer, easy to score objectively, and provide valuable statistical insights regarding class performance on specific questions, helping to assess whether a question was contextually appropriate. 20 22 A standard MCQ consists of a stem, options, and sometimes additional information. 2 23 The stem provides the context, content, and sets up the question, while the options include one correct answer and several incorrect ones, known as distractors. 24 Distractors are crucial in leading noncompetent candidates away from the correct answer, which is a key feature of a well-constructed question. 3 25 However, the main challenge with the MCQ format is that creating high-quality questions is often difficult, time-consuming, and costly. 26

The introduction of ChatGPT, an AI-powered chatbot, has significantly changed the role of AI in education. Trained on a diverse dataset, ChatGPT can perform tasks such as writing songs or poems, telling stories, creating lists, and even creating MCQ exams. 27

The analysis of AI-generated and human expert-generated MCQs in this study provides important insights into their effectiveness in medical education. The KF-20 score for the human experts and AI indicates that the MCQs by human experts are more reliable (KF-20 = 0.67), showing internal consistency, and are better than AI (KF-20 = 0.57).

## Comparable Difficulty

The findings revealed that the difficulty index of AI-generated MCQs (mean = 0.62, SD = 0.14) was similar to that of human expert-generated MCQs (mean = 0.60, SD = 0.13), with no statistically significant difference ( *p* = 0.45). This indicates that AI can produce questions with a difficulty level comparable to those created by human experts. This is a promising outcome for the potential use of AI in generating large question banks for formative assessments, as it suggests that AI can create questions that are appropriately challenging for the target audience. This capability could be particularly useful in contexts where a large volume of questions is needed quickly, such as for practice exams or preparatory materials.

## Practical Applications for Educators

Rather than viewing AI-generated MCQs as replacements for human-authored content, educators can leverage them as a foundational tool. AI can rapidly generate a variety of question stems and plausible distractors, saving time during the initial drafting phase. Educators can then refine these questions by improving distractor quality, aligning content with learning objectives, and enhancing clarity. This hybrid approach balances the efficiency of AI with the pedagogical insight of experienced educators, particularly useful in large-scale formative assessments or low-stakes quizzes where time and resource constraints are prevalent.

## Limitations in Discrimination and Distractor Efficiency

However, the study revealed notable limitations in AI-generated MCQs, particularly regarding the **discrimination index** and DE.

The **discrimination index** , which measures how well a question distinguishes between high- and low-performing students, was significantly higher for expert-generated MCQs (mean = 0.34, SD = 0.08) compared to those generated by AI (mean = 0.28, SD = 0.09; *p* = 0.02). This suggests that human experts are more adept at crafting questions that effectively assess students' depth of understanding and differentiate varying levels of academic performance. The superior performance of expert-generated items may be attributed to the nuanced knowledge of subject matter experts and their awareness of common student misconceptions, enabling them to design questions that better evaluate conceptual understanding.

Similarly, DE, which gauges the plausibility and effectiveness of the incorrect answer choices, was significantly higher in expert-crafted MCQs (mean = 85%, SD = 5%) than in AI-generated ones (mean = 78%, SD = 7%; *p* = 0.01). Human-designed distractors tend to be more contextually appropriate and cognitively challenging, thereby enhancing the diagnostic value of the question by testing not just recall but higher-order thinking and application.

## Why Are Human-Generated MCQs Superior?

The superiority of human-generated MCQs in terms of discrimination and DE can be attributed to several factors:

*Deep understanding of the subject matter* : Human experts have a comprehensive understanding of the intricacies of the subject they are assessing. This allows them to design questions that target specific learning objectives and test higher-order thinking skills, which AI, with its current capabilities, struggles to replicate.

*Insight into student learning behavior* : Human experts are familiar with the common pitfalls and misconceptions that students might have. This knowledge enables them to create distractors that are more likely to mislead students who do not fully understand the material, thereby effectively distinguishing between different levels of student knowledge.

*Pedagogical expertise* : Human experts are trained in educational theory and practice, which informs their ability to construct questions that align with the intended learning outcomes. This pedagogical expertise allows them to design assessments that are not only challenging but also educational, reinforcing learning even as they test it.

*Adaptability* : Human experts can adapt their question-writing strategies based on the evolving needs of the curriculum and the specific challenges faced by students. AI, on the other hand, relies on pre-existing data and algorithms, which may not always capture the nuances of a changing educational environment.

## Implications for Medical Education

The findings of this study suggest that AI-generated MCQs can be valuable, but they are most effective when used as a supportive tool under the guidance of trained educators. The lower discrimination index and DE in AI-generated questions indicate that these tools are not yet capable of fully replicating the expertise and insight that human educators bring to the table. As such, the role of human experts remains crucial, particularly in areas that require a deep understanding of pedagogy and student learning behaviors.

In conclusion, while AI holds promise as a supplementary tool in medical education, particularly for generating large volumes of practice questions, it cannot yet replace the nuanced and expert-driven process of question creation that human educators provide. The findings emphasize the continued importance of human involvement in the assessment process to ensure that evaluations are both fair and effective in measuring student learning outcomes.

**Limitations:** Single-institution study: This study was conducted at a single institution, limiting the generalizability of the findings. The educational environment, student population, and curriculum at one medical college may not be fully representative of other medical colleges of India or globally.

**Scope of AI tools:** Only three AI tools were used in this study, and they were tested on a limited set of topics. Future research may be used to explore a wider range of AI tools and include a broader array of topics to determine whether the findings are consistent across different areas of medical education. Despite the promise of AI in educational assessment, several limitations warrant consideration. First, students' familiarity or lack thereof with AI-generated content may influence their engagement and perceived credibility of the questions. Second, variations among different AI tools, each with unique training data, algorithms, and sensitivity to prompts, can lead to inconsistencies in output quality. Finally, linguistic issues such as awkward phrasing, unnecessarily complex syntax, or culturally unfamiliar references may impair comprehension, particularly for nonnative English speakers.

These findings emphasize the importance of continued human oversight in the integration of AI tools into assessment design, ensuring both reliability and educational value.

## Conclusion

This study provides valuable insights into the potential and limitations of AI-generated MCQs in medical education. While AI tools show promise, particularly in generating questions of appropriate difficulty, human expertise remains essential in crafting high-quality assessments that effectively differentiate between levels of student performance and challenge students' critical thinking. As AI technology continues to evolve, ongoing research and careful implementation will be essential in ensuring that AI contributes positively to medical education.

## References

[1] Cristianini N Intelligence reinvented New Scientist 2016 232 37 41

[2] Gugerty L Newell and Simon's logic theorist: historical background and impact on cognitive modeling Proc Hum Factors Ergon Soc Annu Meet 2006 50 880 884

[3] Weizenbaum J 1966, 9:36 45

[4] Copeland B J MYCIN. Encyclopedia Britannica. November 21, 2018. Accessed October 22, 2025 at:https://www.britannica.com/technology/MYCIN

[5] Accessed October 22, 2025 at:https://www.geeksforgeeks.org/applications-of-ai/

[6] Harry A Role of AI in education INJURUTY 2023 2 260 268

[7] Crompton H Bernacki M Greene J A Psychological foundations of emerging technologies for teaching and learning in higher education Curr Opin Psychol 2020 36 101 105 32604064 10.1016/j.copsyc.2020.04.011

[8] Crompton H Burke D Artificial intelligence in higher education: the state of the field Int J Educ Technol High Educ 2023 20 22

[9] Mir M M Mir G M Raina N T Application of artificial intelligence in medical education: current scenario and future perspectives J Adv Med Educ Prof 2023 11 03 133 140 37469385 10.30476/JAMP.2023.98655.1803 PMC10352669

[10] Sawand F A Chandio B A Bilal M Rasheed M R Raza M A Ahmad N Quality assessment in higher education Intern Let Soc Hum Sci 2023 50 162 171

[11] https://www.watermarkinsights.com/resources/blog/importance-of-assessment-in-higher-education

[12] Nair G G Feroze M Effectiveness of multiple-choice questions (MCQS) discussion as a learning enhancer in conventional lecture class of undergraduate medical students Medical Journal of Dr. D.Y. Patil Vidyapeeth 2023 16 02 S183 S188

[13] Roediger H L Karpicke J D Test-enhanced learning: taking memory tests improves long-term retention Psychol Sci 2006 17 03 249 255 16507066 10.1111/j.1467-9280.2006.01693.x

[14] Nicol D E-assessment by design: using multiple-choice tests to good effect J Furth High Educ 2007 31 53 64

[15] Xhaferi B Xhaferi G Enhancing learning through reflection - a case study of SEEU SEEU Review 2016 12 53 68

[16] Al-Rukban M O Guidelines for the construction of multiple choice questions tests J Family Community Med 2006 13 03 125 133 23012132 PMC3410060

[17] Maheen F Asif M Ahmad H Automatic computer science domain multiple-choice questions generation based on informative sentences PeerJ Comput Sci 2022 8 e1010 10.7717/peerj-cs.1010 PMC9454961 36091982

[18] Kıyak Y S Coşkun Ö Budakoğlu Iİ Uluoğlu C ChatGPT for generating multiple-choice questions: evidence on the use of artificial intelligence in automatic item generation for a rational pharmacotherapy exam Eur J Clin Pharmacol 2024 80 05 729 735 38353690 10.1007/s00228-024-03649-x

[19] Ngo A Gupta S Perrine O Reddy R Ershadi S Remick D ChatGPT 3.5 fails to write appropriate multiple choice practice exam questions Acad Pathol 2023 11 01 100099 38162414 10.1016/j.acpath.2023.100099 PMC10753050

[20] Haladyna T M Downing S M Rodriguez M C A review of multiple-choice item-writing guidelines for classroom assessment Appl Meas Educ 2002 15 309 333

[21] Dion V St-Onge C Bartman I Touchie C Pugh D Written-based progress testing: a scoping review Acad Med 2022 97 05 747 757 34753858 10.1097/ACM.0000000000004507

[22] Iñarrairaegui M Fernández-Ros N Lucena F Evaluation of the quality of multiple-choice questions according to the students' academic level BMC Med Educ 2022 22 01 779 2022 36369070 10.1186/s12909-022-03844-3 PMC9652897

[23] Vegada B Shukla A Khilnani A Charan J Desai C Comparison between three option, four option and five option multiple choice question tests for quality parameters: a randomized study Indian J Pharmacol 2016 48 05 571 575 27721545 10.4103/0253-7613.190757 PMC5051253

[24] Gierl M J Bulut O Guo Q Zhang X Developing, analyzing, and using distractors for multiple-choice tests in education: a comprehensive review Rev Educ Res 2017 87 1082 1116

[25] Kumar D Jaipurkar R Shekhar A Sikri G Srinivas V Item analysis of multiple choice questions: a quality assurance test for an assessment tool Med J Armed Forces India 2021 77 01 S85 S89 33612937 10.1016/j.mjafi.2020.11.007 PMC7873707

[26] Epstein R M Assessment in medical education N Engl J Med 2007 356 04 387 396 17251535 10.1056/NEJMra054784

[27] Kung T H Cheatham M Medenilla A Performance of ChatGPT on USMLE: potential for AI-assisted medical education using large language models PLOS Digit Health 2023 2 02 e0000198 36812645 10.1371/journal.pdig.0000198 PMC9931230

