# Supplementary material for: Human Expertise Outperforms Artificial Intelligence in Medical Education Assessments: MCQ Creation Highlights the Irreplaceable Role of Teachers
Source: Avicenna J Med. 2025 Nov 19;15(4):149–56. doi: 10.1055/s-0045-1813040 (PMC12747746; doi:10.1055/s-0045-1813040)
Supplement: Supplementary file 1 — Supplementary Material [file 10-1055-s-0045-1813040-s250017.pdf]

Supplementary File 01: For Full Prompt

You are a medical physiology expert. You are designing a set of MCQs to test. A total of 50 medical students received a set. Of these, 25 MCQs were made by AI. Using the item response theory (IRT) framework, we calculated key metrics like item reliability, difficulty index, discrimination index, and distractor functionality. Assessing their knowledge to see if they are admitted to basic medical teaching is important, ultimately leading to a specialty level. I have uploaded a sample set of MCQs (sample MCQs) to help you understand their format. Please follow the sample set strictly. There are a few rules to follow when generating the MCQ. I have uploaded a file titled “Tips for MCQ for PEEM” to help you. Importantly, avoid prompting the examinees by using the same or similar words in the question and the answer choice. The category of the question must follow a pre-set blueprint to ensure that a wide range of knowledge is tested. Please refer to the uploaded “blueprint” file. Please make sure that no more than one question is tested on the same condition or organ.

Please review the previously generated MCQ to avoid duplication. Please generate them one by one. For example: I prompt, “Q001” Your response: “Title: Q001 Category: Cardiovascular Disorders, Physiology (Q001) generate MCQs AI said: Perfect, I’ll follow your exact instructions and create one MCQ at a time in line with your blueprint, “Tips for MCQ for PEEM,” and sample format and make sure the questions are validated-style, non-repetitive, and without cueing by repeating words from the stem in the answer choices.

Title: Q001
Category: Cardiovascular Disorders and Physiology
Question: During dynamic exercise, which of the following physiological responses is most responsible for the observed increase in systolic blood pressure?

A. Increased stroke volume due to enhanced venous return
B. Decreased peripheral resistance from vasodilation in skeletal muscles
C. Reduced heart rate secondary to baroreceptor stimulation
D. Increased vagal tone on the sinoatrial node

Correct answer: A. Increased stroke volume due to enhanced venous return
Explanation (for validation, not shown to students): Dynamic exercise augments venous return through the muscle pump and respiratory pump mechanisms. This increases stroke volume, leading to a rise in systolic blood pressure. Although vasodilation decreases total peripheral resistance, this primarily influences diastolic pressure. Heart rate increases (not decreases), and vagal tone is reduced during exercise.

Title: Q001

Category: Respiratory System and Physiology
Question:

During exposure to high altitude, which of the following mechanisms primarily accounts for the initial increase in ventilation?

A. Direct stimulation of central chemoreceptors by low arterial oxygen
B. Reduced bicarbonate reabsorption in the kidneys
C. Activation of peripheral chemoreceptors in the carotid bodies
D. Increased sensitivity of pulmonary stretch receptors

Correct answer: C. Activation of peripheral chemoreceptors in the carotid bodies
Explanation (for validation, not shown to students):
At high altitude, the fall in arterial oxygen tension is detected mainly by peripheral chemoreceptors in the carotid bodies, which stimulate an increase in ventilation. Central chemoreceptors primarily respond to CO_2 changes, not hypoxia. Renal bicarbonate adjustment occurs later as compensation, and pulmonary stretch receptors influence lung inflation reflexes rather than altitude-induced hyperventilation.

Title: Q002
Category: Endocrine System and Physiology
Question:
Which of the following best explains why patients with untreated type 1 diabetes mellitus often present with polyuria?

A. Increased secretion of aldosterone leading to enhanced sodium excretion
B. Osmotic diuresis caused by high concentrations of glucose in the renal filtrate
C. Direct inhibition of antidiuretic hormone release from the posterior pituitary
D. Increased glomerular filtration rate due to hyperinsulinemia

Correct answer: B. Osmotic diuresis caused by high concentrations of glucose in the renal filtrate

Explanation (for validation, not shown to students):
In type 1 diabetes mellitus, lack of insulin leads to hyperglycemia. Once the renal threshold for glucose reabsorption is exceeded, glucose remains in the tubular lumen, creating an osmotic gradient that prevents water reabsorption, resulting in polyuria. Aldosterone secretion does not directly cause polyuria here. ADH release is not inhibited primarily, and hyperinsulinemia is not a feature of untreated type 1 diabetes.

Title: Q003
Category: Reproductive System and Physiology
Question:

 © 2025. The Author(s).

Which of the following hormonal changes triggers ovulation during the menstrual cycle?

A. Gradual increase in follicle-stimulating hormone (FSH)
B. Sudden surge of luteinizing hormone (LH)
C. Steady rise in progesterone
D. Decline in estradiol levels

Correct answer: B. Sudden surge of luteinizing hormone (LH)

Explanation (validation only):

The mid-cycle surge in LH secretion, stimulated by sustained high estradiol levels, is responsible for rupture of the dominant follicle and release of the oocyte. FSH does rise, but it is not the direct trigger. Progesterone increases after ovulation, and estradiol levels do not fall immediately before ovulation.

Title: Q004

Category: Nervous System and Physiology

Question:

In an experimental setup, stimulation of the preganglionic sympathetic fibers leads to which of the following neurotransmitters being released at the synapse with postganglionic neurons?

A. Norepinephrine
B. Acetylcholine
C. Dopamine
D. Epinephrine

Correct answer: B. Acetylcholine

Explanation (validation only):

Preganglionic sympathetic neurons release acetylcholine, which acts on nicotinic receptors of postganglionic neurons. Postganglionic sympathetic fibers typically release norepinephrine at target organs (except sweat glands). Epinephrine is mainly secreted by the adrenal medulla.

Title: Q005

Category: Special Senses and Physiology

Question:

Which of the following best describes the physiological basis of the blind spot in the human eye?

A. High density of rods without cones
B. Absence of photoreceptors at the optic disc
C. Overlapping of visual fields from both eyes
D. Saturation of rhodopsin in low light

Correct answer: B. Absence of photoreceptors at the optic disc

Explanation (validation only):

The blind spot corresponds to the optic disc, where ganglion cell axons exit the eye to form the optic nerve. No rods or cones are present at this site. Rod density is high at the periphery, not at the blind spot. Visual field overlap provides binocular vision, not a blind spot. Rhodopsin saturation relates to dark adaptation.

Title: Q006

Category: Gastrointestinal System and Physiology

Question:

Which of the following gastrointestinal hormones is primarily responsible for stimulating gallbladder contraction?

A. Secretin
B. Cholecystokinin (CCK)
C. Gastrin
D. Motilin

Correct answer: B. Cholecystokinin (CCK)

Explanation (validation only):

CCK, released from the I-cells of the duodenum in response to fatty acids and amino acids, induces gallbladder contraction and relaxation of the sphincter of Oddi. Secretin stimulates bicarbonate secretion, gastrin enhances gastric acid secretion, and motilin regulates interdigestive migrating motor complexes.

Title: Q007

Category: Excretory System and Physiology

Question:

Which segment of the nephron is impermeable to water, contributing to the dilution of tubular fluid?

A. Proximal convoluted tubule
B. Descending limb of Henle's loop
C. Thick ascending limb of Henle's loop
D. Collecting duct in the presence of ADH

Correct answer: C. Thick ascending limb of Henle's loop

Explanation (validation only):

The thick ascending limb actively reabsorbs sodium, potassium, and chloride but is impermeable to water, leading to dilution of tubular fluid. The proximal tubule and descending limb allow water movement. Collecting duct permeability depends on ADH presence.

Title: Q008

Category: Blood and Immune System and Physiology

Question:

Which clotting factor is vitamin K–dependent and directly involved in the conversion of prothrombin to thrombin?

A. Factor VIII
B. Factor IX
C. Factor X
D. Factor XII

Correct answer: C. Factor X

Explanation (validation only):

Activated factor X (Xa), together with factor V, calcium, and phospholipids, forms the prothrombinase complex, converting prothrombin into thrombin. Factors IX and VII are also vitamin K–dependent but do not directly form the prothrombinase complex. Factor VIII is part of the intrinsic pathway, and factor XII initiates the contact pathway.

 © 2025. The Author(s).

Title: Q009
Category: Nerve–Muscle and Physiology
Question:
At the neuromuscular junction, acetylcholine binding to nicotinic receptors leads to which immediate effect?

A. Inhibition of voltage-gated sodium channels
B. Generation of an end-plate potential due to sodium influx
C. Blockade of potassium efflux from the motor end plate
D. Release of calcium from the sarcoplasmic reticulum

Correct answer: B. Generation of an end-plate potential due to sodium influx
Explanation (validation only):
When acetylcholine binds to nicotinic receptors at the motor end plate, it opens ligand-gated ion channels, allowing sodium influx and producing an end-plate potential. This depolarization triggers action potential propagation, ultimately leading to calcium release and muscle contraction.

Title: Q010
Category: Cardiovascular System and Physiology
Question:
Which of the following best explains the dicrotic notch seen in the aortic pressure waveform?

A. Closure of the mitral valve during isovolumetric contraction
B. Elastic recoil of the aorta during systole
C. Backward flow of blood causing closure of the aortic valve
D. Forward ejection of blood during ventricular systole

Correct answer: C. Backward flow of blood causing closure of the aortic valve
Explanation (validation only):
The dicrotic notch (incisura) represents a brief rise in aortic pressure after the closure of the aortic valve, due to transient backward flow of blood. Mitral valve events are unrelated. Elastic recoil sustains diastolic pressure but does not directly cause the notch.

Title: Q011
Category: Respiratory System and Physiology
Question:
Which of the following occurs during obstructive lung disease, such as asthma?

A. Increased FEV1/FVC ratio
B. Reduced residual volume
C. Increased airway resistance
D. Decreased total lung capacity

Correct answer: C. Increased airway resistance
Explanation (validation only):
Asthma causes narrowing of the airways, leading to increased resistance. FEV1/FVC ratio decreases (not increases). Residual volume is increased due to air trapping, and total lung capacity may remain normal or increase, not decrease.

Title: Q012
Category: Endocrine System and Physiology
Question:
Which hormone is primarily responsible for stimulating gluconeogenesis during prolonged fasting?

A. Insulin
B. Cortisol
C. Thyroxine
D. Calcitonin

Correct answer: B. Cortisol
Explanation (validation only):
Cortisol enhances gluconeogenesis by increasing the expression of key enzymes in the liver. Insulin inhibits gluconeogenesis. Thyroxine increases basal metabolic rate but does not directly regulate gluconeogenesis. Calcitonin regulates calcium homeostasis.

Title: Q013
Category: Reproductive System and Physiology
Question:
Which of the following prevents multiple follicles from ovulating during a single menstrual cycle?

A. High levels of progesterone suppressing FSH
B. Inhibin produced by granulosa cells suppressing FSH secretion
C. Estradiol stimulating the LH surge
D. Oxytocin inhibiting follicular maturation

Correct answer: B. Inhibin produced by granulosa cells suppressing FSH secretion
Explanation (validation only):
Inhibin from granulosa cells inhibits FSH release, preventing maturation of additional follicles once the dominant follicle is selected. Progesterone acts later, estradiol promotes LH surge, and oxytocin does not regulate folliculogenesis.

Title: Q014
Category: Nervous System and Physiology
Question:
Which ion channel is primarily responsible for the rapid depolarization phase of a neuronal action potential?

A. Voltage-gated sodium channel
B. Voltage-gated potassium channel
C. Calcium-activated chloride channel
D. Ligand-gated potassium channel

Correct answer: A. Voltage-gated sodium channel
Explanation (validation only):

The opening of voltage-gated sodium channels allows Na^+ influx, producing rapid depolarization. Potassium channels mediate repolarization. Calcium-activated chloride and ligand-gated potassium channels are not the main drivers of depolarization.

 © 2025. The Author(s).

Title: Q015
Category: Special Senses and Physiology
Question:
Which of the following best explains the ability of the lens to increase its curvature during accommodation for near vision?

A. Contraction of extraocular muscles
B. Contraction of ciliary muscles reducing tension on the suspensory ligaments
C. Relaxation of ciliary muscles increasing zonular tension
D. Inhibition of parasympathetic activity to the ciliary body

Correct answer: B. Contraction of ciliary muscles reducing tension on the suspensory ligaments
Explanation (validation only):
Parasympathetic stimulation contracts the ciliary muscle, releasing tension on the suspensory ligaments (zonules), allowing the elastic lens to become more convex for near vision. Extraocular muscles do not alter lens shape.

Title: Q016
Category: Gastrointestinal System and Physiology
Question:
Which of the following factors most strongly stimulates gastric acid secretion during the cephalic phase of digestion?

A. Direct action of fatty acids on G cells
B. Vagal stimulation via acetylcholine and gastrin-releasing peptide
C. Local irritation of the gastric mucosa
D. Secretin release from the duodenum

Correct answer: B. Vagal stimulation via acetylcholine and gastrin-releasing peptide
Explanation (validation only):
The cephalic phase is initiated by the sight, smell, and thought of food, mediated through vagal stimulation. Fatty acids act later in the intestinal phase. Local irritation may stimulate protective reflexes, not acid secretion. Secretin inhibits gastric acid secretion.

Title: Q017
Category: Excretory System and Physiology
Question:
Which of the following best explains why administration of a loop diuretic such as furosemide increases urinary calcium excretion?

A. Inhibition of sodium-glucose cotransporter in the proximal tubule
B. Reduced paracellular reabsorption of calcium in the thick ascending limb
C. Blockade of aldosterone receptors in the collecting duct
D. Increased ADH action on the distal nephron

Correct answer: B. Reduced paracellular reabsorption of calcium in the thick ascending limb
Explanation (validation only):
Loop diuretics inhibit the Na^+–K^+–$2Cl^-$ symporter in the thick ascending limb, reducing the positive transepithelial potential that normally drives calcium reabsorption, thus increasing calcium excretion. Aldosterone blockade occurs with potassium-sparing diuretics, not loop diuretics.

Title: Q018
Category: Blood and Immune System and Physiology
Question:
Which of the following immune cells is the primary mediator of the delayed-type (Type IV) hypersensitivity reaction?

A. Neutrophils
B. $CD4^+$ T lymphocytes
C. Mast cells
D. B lymphocytes

Correct answer: B. $CD4^+$ T lymphocytes
Explanation (validation only):
Type IV hypersensitivity (delayed type) is mediated by activated $CD4^+$ T-helper cells releasing cytokines that recruit macrophages. Neutrophils dominate acute inflammation, mast cells mediate immediate hypersensitivity (Type I), and B cells are key in antibody-mediated (Type II/III) reactions.

Title: Q019
Category: Nerve–Muscle and Physiology
Question:
Which of the following best explains the latent period observed between the application of a single stimulus and the onset of muscle contraction?

A. Time required for cross-bridge cycling
B. Delay due to calcium release from the sarcoplasmic reticulum
C. Inhibition of acetylcholinesterase activity
D. Refractory period of the action potential

Correct answer: B. Delay due to calcium release from the sarcoplasmic reticulum
Explanation (validation only):
The latent period represents the time needed for excitation–contraction coupling: depolarization, calcium release from the sarcoplasmic reticulum, and troponin binding. Cross-bridge cycling occurs after this phase. The refractory period refers to neuronal or muscle excitability, not the latent delay.

Title: Q020
Category: Cardiovascular System and Physiology
Question:
Which of the following changes is most likely to occur during prolonged standing without movement (orthostatic stress)?

A. Increased venous return and stroke volume
B. Decreased cardiac output due to venous pooling

 © 2025. The Author(s).

C. Reflex bradycardia from carotid baroreceptor activation
D. Increased preload due to skeletal muscle contraction

Correct answer: B. Decreased cardiac output due to venous pooling
Explanation (validation only):
Prolonged standing causes blood pooling in the lower extremities, reducing venous return, stroke volume, and cardiac output. Reflex tachycardia, not bradycardia, occurs. Skeletal muscle contraction would improve venous return, but it is absent during immobility.

Title: Q021
Category: Respiratory System and Physiology
Question:
Which lung volume cannot be measured directly by spirometry?

A. Tidal volume
B. Inspiratory reserve volume
C. Residual volume
D. Expiratory reserve volume

Correct answer: C. Residual volume
Explanation (validation only):
Residual volume (the air remaining after maximal expiration) cannot be measured by simple spirometry, as it requires helium dilution or body plethysmography. Tidal, inspiratory reserve, and expiratory reserve volumes are all directly measurable.

Title: Q022
Category: Endocrine System and Physiology
Question:
Which of the following hormones exerts its action primarily through a cytoplasmic receptor that translocates to the nucleus?

A. Epinephrine
B. Cortisol
C. Glucagon
D. Insulin

Correct answer: B. Cortisol
Explanation (validation only):
Cortisol, a steroid hormone, binds to cytoplasmic receptors, which then move to the nucleus to regulate gene transcription. Epinephrine and glucagon act via membrane receptors and second messengers (cAMP). Insulin acts through a receptor tyrosine kinase.

Title: Q023
Category: Reproductive System and Physiology
Question:
Which of the following is the primary physiological effect of progesterone during the luteal phase of the menstrual cycle?

A. Proliferation of endometrial glands
B. Secretory transformation of the endometrium
C. Initiation of the LH surge
D. Stimulation of follicular development

Correct answer: B. Secretory transformation of the endometrium
Explanation (validation only):
Progesterone secreted by the corpus luteum promotes glandular secretion and stromal changes in the endometrium, preparing it for implantation. Estradiol drives proliferation before ovulation. LH surge occurs before ovulation, not in the luteal phase.

Title: Q024
Category: Nervous System and Physiology
Question:
Which neurotransmitter is primarily involved in long-term potentiation (LTP) in the hippocampus, a mechanism for memory formation?

A. GABA
B. Dopamine
C. Glutamate
D. Serotonin

Correct answer: C. Glutamate
Explanation (validation only):
Glutamate activates NMDA and AMPA receptors in hippocampal neurons, playing a central role in long-term potentiation and memory. GABA is inhibitory, dopamine and serotonin modulate learning, but are not the primary transmitters in LTP.

Title: Q025
Category: Special Senses and Physiology
Question:
Which of the following structures is responsible for detecting angular acceleration of the head?

A. Cochlea
B. Utricle
C. Saccule
D. Semi-circular canals

Correct answer: D. Semi-circular canals
Explanation (validation only):
The semi-circular canals contain ampullary cristae that detect angular acceleration. The utricle and saccule detect linear acceleration and gravity. The cochlea detects sound, not head movement.

Title: Q026
Category: Gastrointestinal System and Physiology
Question:
Which of the following best explains the alkaline tide observed after a meal?

A. Increased secretion of bicarbonate into the pancreatic ducts
B. Entry of bicarbonate into venous blood as gastric parietal cells secrete HCl

C. Reduced bile acid reabsorption from the ileum
D. Secretion of bicarbonate by Brunner's glands

Correct answer: B. Entry of bicarbonate into venous blood as gastric parietal cells secrete HCl
Explanation (validation only):
When parietal cells secrete hydrogen ions into the stomach lumen, bicarbonate is simultaneously released into venous blood, producing a transient postprandial rise in plasma bicarbonate (alkaline tide). Pancreatic and Brunner's gland secretions contribute bicarbonate to the lumen but not to the blood.

Title: Q027
Category: Excretory System and Physiology
Question:
Which of the following best explains the counter-current multiplier mechanism in the loop of Henle?

A. It increases glomerular filtration rate
B. It generates a hyperosmotic medullary interstitium
C. It maintains a constant tubular sodium concentration
D. It prevents reabsorption of urea in the inner medulla

Correct answer: B. It generates a hyperosmotic medullary interstitium
Explanation (validation only):
The counter-current multiplier created by the loop of Henle establishes a gradient of increasing osmolality in the medullary interstitium, essential for urine concentration. It does not alter GFR directly, sodium concentration varies along the nephron, and urea recycling contributes to (not prevents) medullary hypertonicity.

Title: Q028
Category: Blood and Immune System and Physiology
Question:
Which of the following accurately describes the role of transferrin in blood?

A. It stores iron within macrophages
B. It transports iron in plasma to developing erythrocytes
C. It catalyzes heme synthesis in red cells
D. It promotes intestinal absorption of dietary iron

Correct answer: B. It transports iron in plasma to developing erythrocytes
Explanation (validation only):
Transferrin binds ferric iron (Fe^{3+}) in plasma and delivers it to erythroid precursors in the bone marrow. Ferritin stores iron, heme synthesis requires enzymes, and intestinal absorption is regulated mainly by ferroportin and hepcidin.

Title: Q029
Category: Nerve–Muscle and Physiology
Question:
Which of the following occurs during the isometric phase of a muscle contraction?

A. The muscle shortens while tension remains constant
B. Cross-bridge cycling generates force without changing muscle length
C. Sarcomere length decreases rapidly during contraction
D. Tension falls below the load applied to the muscle

Correct answer: B. Cross-bridge cycling generates force without changing muscle length
Explanation (validation only):
In isometric contraction, the cross-bridges form and cycle, generating force, but the muscle length does not change because the load is not moved. In isotonic contraction, the muscle shortens at constant tension.

Title: Q030
Category: Cardiovascular System and Physiology
Question:
Which of the following changes is most likely to occur immediately after a sudden hemorrhage of 1 liter of blood?

A. Increased arterial baroreceptor firing
B. Reflex tachycardia due to sympathetic activation
C. Increased renal perfusion pressure
D. Increased venous return to the right atrium

Correct answer: B. Reflex tachycardia due to sympathetic activation
Explanation (validation only):
Acute blood loss decreases arterial pressure, reducing baroreceptor firing. This activates the sympathetic system, causing tachycardia and vasoconstriction. Renal perfusion and venous return fall, not rise.

Title: Q031
Category: Respiratory System and Physiology
Question:
Which of the following best explains why carbon monoxide poisoning reduces oxygen delivery to tissues?

A. CO binds to the same site as oxygen on hemoglobin with greater affinity
B. CO stimulates hyperventilation causing respiratory alkalosis
C. CO reduces 2,3-BPG levels in red blood cells
D. CO directly inhibits mitochondrial ATP synthase

Correct answer: A. CO binds to the same site as oxygen on hemoglobin with greater affinity
Explanation (validation only):
Carbon monoxide binds hemoglobin with $\sim$200 to 250 times the affinity of oxygen, displacing oxygen and reducing oxygen delivery. It also shifts the dissociation curve leftward, impairing unloading. Hyperventilation and ATP synthase inhibition are not the primary mechanisms.

Title: Q032
Category: Endocrine System, Physiology
Question:
Which of the following best explains why parathyroid hormone (PTH) increases plasma calcium concentration?

 © 2025. The Author(s).

A. Inhibition of osteoclast activity
B. Increased renal excretion of calcium
C. Enhanced bone resorption and stimulation of calcitriol formation
D. Increased secretion of calcitonin from thyroid C cells

Correct answer: C. Enhanced bone resorption and stimulation of calcitriol formation
Explanation (validation only):
PTH increases calcium levels by stimulating osteoclast-mediated bone resorption (via osteoblast signaling), enhancing renal calcium reabsorption, and increasing calcitriol formation to promote intestinal calcium absorption. It does not inhibit osteoclasts or calcitonin.

Title: Q033
Category: Reproductive System and Physiology
Question:
Which of the following events is essential for the maintenance of early pregnancy before placental takeover?

A. Secretion of human chorionic gonadotropin (hCG) by the trophoblast
B. Decline in progesterone secretion from the corpus luteum
C. Increased secretion of FSH from the anterior pituitary
D. Rapid rise in maternal prolactin levels

Correct answer: A. Secretion of human chorionic gonadotropin (hCG) by the trophoblast
Explanation (validation only):
hCG maintains the corpus luteum, which continues progesterone production until the placenta takes over. A decline in progesterone would terminate pregnancy. FSH and prolactin do not maintain early pregnancy.

Title: Q034
Category: Nervous System and Physiology
Question:
Which of the following receptors is primarily responsible for detecting muscle stretch and initiating the stretch reflex?

A. Golgi tendon organ
B. Muscle spindle
C. Pacinian corpuscle
D. Ruffini endings

Correct answer: B. Muscle spindle
Explanation (validation only):
Muscle spindles detect changes in muscle length and mediate the stretch reflex. Golgi tendon organs detect tension and prevent excessive force. Pacinian corpuscles sense vibration, and Ruffini endings detect sustained pressure.

Title: Q035
Category: Special Senses and Physiology
Question:
Which of the following best explains why vitamin A deficiency can cause night blindness?

A. Impaired regeneration of rhodopsin in rod cells
B. Loss of cone cell function in the fovea
C. Degeneration of the optic nerve
D. Reduced aqueous humor production

Correct answer: A. Impaired regeneration of rhodopsin in rod cells
Explanation (validation only):
Vitamin A (retinol) is required for the synthesis of retinal, a component of rhodopsin. Without it, rods cannot regenerate rhodopsin, leading to impaired vision in dim light. Cones are less affected, optic nerve degeneration is unrelated, and aqueous humor production involves ciliary body function.

Title: Q036
Category: Gastrointestinal System and Physiology
Question:
Which of the following best explains why pancreatic enzymes are secreted in inactive forms?

A. To prevent the digestion of bile salts in the gallbladder
B. To avoid autodigestion of pancreatic tissue
C. To inhibit gastric acid secretion in the stomach
D. To delay protein digestion until absorption

Correct answer: B. To avoid autodigestion of pancreatic tissue
Explanation (validation only):
Pancreatic enzymes (e.g., trypsinogen, chymotrypsinogen) are secreted as zymogens to prevent self-digestion of the pancreas. They are activated in the small intestine by enteropeptidase. Bile salts are not digested, gastric acid secretion is not regulated by enzymes, and protein digestion begins in the stomach.

Title: Q037
Category: Excretory System and Physiology
Question:
In the absence of antidiuretic hormone (ADH), which portion of the nephron remains impermeable to water, resulting in dilute urine?

A. Proximal convoluted tubule
B. Descending limb of Henle's loop
C. Thick ascending limb of Henle's loop
D. Collecting duct

Correct answer: D. Collecting duct
Explanation (validation only):
Without ADH, the collecting duct is impermeable to water, so water cannot be reabsorbed, leading to dilute urine. The proximal tubule and descending limb are always water-permeable, while the thick ascending limb is impermeable regardless of ADH.

Title: Q038
Category: Blood and Immune System and Physiology
Question:

Which of the following best explains the prolonged bleeding time observed in patients with von Willebrand disease?

A. Deficiency of platelet-derived growth factor
B. Defective platelet adhesion to subendothelial collagen
C. Absence of thrombin generation
D. Excess fibrinolytic activity in plasma

Correct answer: B. Defective platelet adhesion to subendothelial collagen
Explanation (validation only):
von Willebrand factor is necessary for platelet adhesion to collagen at sites of vascular injury. Its deficiency or dysfunction impairs platelet plug formation, prolonging bleeding time. Thrombin generation, fibrinolysis, and PDGF are not the primary defects.

Title: Q039
Category: Nerve–Muscle and Physiology
Question:
Which of the following best describes the role of troponin C in skeletal muscle contraction?

A. Hydrolyzes ATP to provide energy for cross-bridge cycling
B. Binds calcium to initiate conformational change in tropomyosin
C. Anchors myosin to the Z-line of the sarcomere
D. Provides structural elasticity to sarcomeres

Correct answer: B. Binds calcium to initiate conformational change in tropomyosin
Explanation (validation only):
Troponin C binds calcium, causing tropomyosin to shift and expose myosin-binding sites on actin, allowing cross-bridge formation. ATP hydrolysis is performed by myosin ATPase. Titin (not troponin) provides elasticity and anchors myosin to the Z-line.

Title: Q040
Category: Cardiovascular System and Physiology
Question:
Which of the following best explains why pulse pressure increases during exercise?

A. Rise in diastolic pressure due to vasoconstriction
B. Increase in stroke volume from enhanced venous return
C. Reduced cardiac contractility
D. Decreased systolic ejection velocity

Correct answer: B. Increase in stroke volume from enhanced venous return
Explanation (validation only):
Exercise augments venous return and myocardial contractility, increasing stroke volume, which raises systolic pressure and widens pulse pressure. Diastolic pressure may remain stable or fall slightly due to vasodilation. Contractility increases, not decreases, and systolic ejection velocity rises.

Title: Q041
Category: Respiratory System and Physiology
Question:
Which of the following best explains the increase in ventilation during moderate exercise?

A. Stimulation of central chemoreceptors by hypoxemia
B. Reflexes from muscle and joint proprioceptors
C. Activation of J-receptors due to pulmonary congestion
D. Direct hypoxic stimulation of medullary neurons

Correct answer: B. Reflexes from muscle and joint proprioceptors
Explanation (validation only):
During moderate exercise, ventilation increases primarily due to feed-forward signals from proprioceptors and central command. Hypoxemia does not occur, J-receptors are not activated in normal exercise, and medullary neurons respond to chemical changes, not direct hypoxia.

Title: Q042
Category: Endocrine System and Physiology
Question:
Which of the following is the primary action of insulin on skeletal muscle cells?

A. Activation of lipolysis
B. Stimulation of glucose uptake via GLUT-4 transporters
C. Inhibition of protein synthesis
D. Stimulation of glycogen breakdown

Correct answer: B. Stimulation of glucose uptake via GLUT-4 transporters
Explanation (validation only):
Insulin increases glucose uptake in skeletal muscle by translocating GLUT-4 transporters to the plasma membrane. It promotes glycogen synthesis and protein synthesis, while inhibiting lipolysis.

Title: Q043
Category: Reproductive System and Physiology
Question:
Which of the following best explains the acrosome reaction in human fertilization?

A. Penetration of the zona pellucida by the release of hydrolytic enzymes
B. Fusion of sperm and oocyte plasma membranes
C. Completion of the first meiotic division in the oocyte
D. Rapid depolarization of the oocyte membrane

Correct answer: A. Penetration of the zona pellucida by release of hydrolytic enzymes
Explanation (validation only):
The acrosome reaction releases enzymes (e.g., hyaluronidase, acrosin) that digest the zona pellucida, allowing sperm entry. Membrane fusion occurs afterward, and meiotic completion and depolarization are subsequent steps in fertilization.

Title: Q044
Category: Nervous System and Physiology
Question:
Which of the following best explains the absolute refractory period of a neuron?

A. Inactivation of voltage-gated sodium channels
B. Opening of ligand-gated potassium channels
C. Depletion of neurotransmitter at the synapse
D. Activation of chloride influx through GABA receptors

Correct answer: A. Inactivation of voltage-gated sodium channels
Explanation (validation only):
During the absolute refractory period, sodium channels are inactivated and cannot reopen until the membrane repolarizes, preventing another action potential. Potassium channels mediate repolarization but do not cause refractoriness. Neurotransmitter depletion and chloride influx are not the primary mechanisms.

Title: Q045
Category: Special Senses and Physiology
Question:
Which of the following best explains the basis of the Weber test in detecting unilateral conductive hearing loss?

A. Sound is perceived louder in the normal ear due to intact cochlear function
B. Sound lateralizes to the affected ear because ambient noise is reduced
C. Bone conduction bypasses the middle ear and stimulates both cochleae equally
D. Sound is perceived louder in the affected ear due to enhanced cochlear sensitivity

Correct answer: B. Sound lateralizes to the affected ear because ambient noise is reduced
Explanation (validation only):
In conductive hearing loss, ambient noise is diminished in the affected ear, so bone-conducted vibrations are perceived more strongly there. In sensorineural loss, sound lateralizes to the normal ear.

Title: Q046
Category: Gastrointestinal System and Physiology
Question:
Which of the following best explains the role of cholecystokinin (CCK) in digestion?

A. Stimulates gastric acid secretion from parietal cells
B. Relaxes the sphincter of Oddi and stimulates gallbladder contraction
C. Enhances secretion of intrinsic factor from the gastric mucosa
D. Inhibits bile salt reabsorption in the ileum

Correct answer: B. Relaxes the sphincter of Oddi and stimulates gallbladder contraction
Explanation (validation only):
CCK is secreted by I-cells in the duodenum in response to fats and proteins. It contracts the gallbladder, relaxes the sphincter of Oddi, and stimulates pancreatic enzyme secretion. It does not directly increase gastric acid or intrinsic factor secretion.

Title: Q047
Category: Excretory System and Physiology
Question:
Which of the following changes occurs when an individual is water-deprived for 24 hours?

A. Decreased plasma osmolality
B. Reduced secretion of antidiuretic hormone (ADH)
C. Increased permeability of the collecting duct to water
D. Decreased medullary interstitial osmotic gradient

Correct answer: C. Increased permeability of the collecting duct to water
Explanation (validation only):
Water deprivation increases plasma osmolality, stimulating ADH secretion. ADH increases water reabsorption in the collecting ducts by inserting aquaporins, leading to concentrated urine. The medullary osmotic gradient is maintained or enhanced, not reduced.

Title: Q048
Category: Blood and Immune System and Physiology
Question:
Which of the following best explains the role of tissue factor (factor III) in hemostasis?

A. Activates factor VII to initiate the extrinsic pathway
B. Converts fibrinogen to fibrin directly
C. Stabilizes fibrin clot by cross-linking
D. Activates plasminogen to plasmin

Correct answer: A. Activates factor VII to initiate the extrinsic pathway
Explanation (validation only):
Tissue factor (thromboplastin) exposed after vascular injury binds and activates factor VII, initiating the extrinsic coagulation pathway. Thrombin converts fibrinogen to fibrin, factor XIII stabilizes the clot, and plasminogen activation is part of fibrinolysis.

Title: Q049
Category: Nerve-Muscle and Physiology
Question:
Which of the following best explains why skeletal muscle contraction is prevented in the absence of extracellular calcium at the neuromuscular junction?

A. Calcium entry is required for synaptic vesicle fusion and acetylcholine release
B. Calcium directly binds to actin to expose binding sites for myosin
C. Calcium activates ATP hydrolysis in myosin heads
D. Calcium entry depolarizes the sarcolemma to initiate action potentials

 © 2025. The Author(s).

Correct answer: A. Calcium entry is required for synaptic vesicle fusion and acetylcholine release

Explanation (validation only):

At the neuromuscular junction, calcium influx through voltage-gated channels triggers vesicle fusion and acetylcholine release. Without extracellular calcium, neurotransmitter release fails, and contraction cannot occur. Intracellular calcium, not extracellular, binds troponin C for cross-bridge cycling.

Title: Q050

Category: Cardiovascular System and Physiology

Question:

Which of the following best explains the dicrotic notch observed in the aortic pressure tracing?

A. Closure of the mitral valve at the onset of systole
B. Elastic recoil of the aorta during diastole
C. Transient backflow of blood causing closure of the aortic valve
D. Opening of the pulmonary valve during right ventricular systole

Correct answer: C. Transient backflow of blood causing closure of the aortic valve

Explanation (validation only):

The dicrotic notch (incisura) is a small upward deflection in the aortic pressure curve, caused by a brief retrograde flow of blood immediately following aortic valve closure. It is not due to mitral or pulmonary valve events, and while elastic recoil maintains diastolic pressure, it does not produce the notch itself.

Supplementary File 02: AI-Generated MCQ and Prompt According to Classification

MCQ 1

Classification table

Specialty	Subspecialty	Disease	Task	Sub-task	Complexity	Level of study
Physiology	Immunophysiology	SLE	Diagnosis	Serology	Three-jump	Senior level

Question Stem

A 28-year-old woman presents with photosensitivity, joint pain, and oral ulcers. On examination, she has an erythematous rash over her cheeks, sparing the nasolabial folds. Laboratory evaluation shows:

Test	Patient value	Normal range (SI units)
ANA	Positive (1:320, speckled)	Negative
Anti-dsDNA	Positive	Negative
Complement C3	0.40 g/L	0.9–1.8 g/L
Complement C4	0.08 g/L	0.1–0.4 g/L

From a **physiological perspective**, the low complement levels observed in this patient are primarily due to:

Options

A. Increased genetic deficiency of complement proteins
B. Accelerated consumption of complement in immune complex formation
C. Decreased hepatic synthesis of complement proteins
D. Direct neutralization of complement by autoantibodies
E. Enhanced urinary excretion of complement proteins

Correct answer: B. Accelerated consumption of complement in immune complex formation.

Explanation:

Correct (B): In SLE, antigen–antibody complexes activate the classical complement pathway. This leads to consumption of C3 and C4, explaining their low serum levels.

A. Genetic deficiency: Rare congenital complement deficiencies predispose to lupus-like disease but are not the cause of complement consumption in acquired SLE.

C. Decreased hepatic synthesis: The liver produces complement normally in SLE; suppression is not typical.

D. Neutralization by autoantibodies: Autoantibodies do not directly inactivate complement proteins; instead, they drive immune complex formation.

E. Urinary excretion: Complement proteins are large molecules and not typically lost in urine even with nephritis.

 © 2025. The Author(s).

Specialty	Subspecialty	Disease	Task	Sub-task	Complexity	Level of study
Physiology	Renal physiology	SLE	Diagnosis	Glomerular filtration	Three-jump	Senior level

Question Stem

A 35-year-old woman with known SLE presents with facial puffiness and ankle oedema. Blood pressure is 155/95 mm Hg. Urinalysis shows 3+ proteinuria and microscopic hematuria.

Laboratory results:

Test	Patient value	Normal range (SI units)
Serum creatinine	2.1 mg/dL (186 µmol/L)	0.6–1.2 mg/dL (53–106 µmol/L)
Serum albumin	2.2 g/dL	3.5–5.0 g/dL
24-h urinary protein	5.2 g	<0.15 g
Complement C3	0.5 g/L	0.9–1.8 g/L

Which physiological mechanism best explains the development of **edema** in this patient?

Options

A. Decreased plasma oncotic pressure due to hypoalbuminemia.
B. Increased hydrostatic pressure due to systemic hypertension.
C. Lymphatic obstruction from immune complex deposition
D. Sodium and water retention from hyperaldosteronism alone
E. Increased vascular permeability due to circulating cytokines only

Correct answer: A. Decreased plasma oncotic pressure due to hypoalbuminemia

Explanation:

Correct (A): Severe proteinuria leads to hypoalbuminemia, reducing plasma oncotic pressure and favoring fluid movement into interstitial spaces → edema.

B. Hydrostatic pressure: Contributes to edema but is not the primary mechanism in nephrotic syndrome.

C. Lymphatic obstruction: Not a feature of SLE nephritis.

D. Aldosterone: Secondary sodium retention worsens oedema, but the initiating mechanism is loss of plasma oncotic pressure.

E. Cytokines: Increase permeability, but in lupus nephritis, hypoalbuminemia dominates.

MCQ 3

Classification table

Specialty	Subspecialty	Disease	Task	Sub-task	Complexity	Level of study
Physiology	Autonomic nervous system	SLE	Diagnosis	Cardiovascular reflex	Three-jump	Senior level

Question Stem

A 29-year-old woman with SLE reports dizziness and palpitations when standing. On examination, BP is 110/70 mm Hg supine and 85/60 mm Hg on standing; heart rate increases from 72 to 105 beats/min.

This abnormal response most likely reflects which physiological dysfunction?

Options

A. Impaired baroreceptor reflex sensitivity due to autonomic dysfunction
B. Reduced cardiac output from pericardial effusion alone
C. Excessive renin release with exaggerated sympathetic activity
D. Direct myocardial suppression by circulating autoantibodies
E. Decreased atrial stretch receptor stimulation due to hypovolemia only

Correct answer: A. Impaired baroreceptor reflex sensitivity due to autonomic dysfunction

Explanation:

Correct (A): Autonomic dysfunction in SLE reduces baroreceptor-mediated compensation, leading to orthostatic hypotension with excessive tachycardia.

B. Pericardial effusion: Causes low cardiac output but not this reflex pattern.

C. Renin release: Causes hypertension, not postural hypotension.

D. Myocardial suppression: May cause heart failure, not exaggerated orthostatic changes.

E. Hypovolemia: Can contribute, but autonomic dysfunction is the primary issue here.

MCQ 4

Classification table

Specialty	Subspecialty	Disease	Task	Sub-task	Complexity	Level of study
Physiology	Immunophysiology	SLE	Diagnosis	Cytokine regulation	Three-jump	Senior level

Question Stem

A 33-year-old woman with SLE has persistent fatigue, low-grade fever, and active arthritis despite immunosuppressive therapy. Serum studies reveal elevated IL-6 and TNF-α levels.

Which physiological effect of IL-6 most directly explains the patient's anemia of chronic disease?

Options

A. Suppression of erythropoietin release from the kidney.
B. Stimulation of hepcidin production in the liver.
C. Direct destruction of red blood cells in circulation.
D. Increased gastrointestinal iron loss due to mucosal damage.
E. Decreased bone marrow stem cell numbers by apoptosis.

Correct answer: B. Stimulation of hepcidin production in the liver

Explanation:

Correct (B): IL-6 stimulates hepatocytes to produce hepcidin → decreased iron absorption and sequestration → anemia of chronic disease.

A. Erythropoietin suppression: Seen in renal failure, not the main mechanism here.

C. RBC destruction: Hemolysis occurs in SLE but not via IL-6.

D. GI iron loss: Not mediated by IL-6.

E. Stem cell apoptosis: Occurs in aplastic anemia, not cytokine-driven anemia of chronic disease.

MCQ 5

Classification table

Specialty	Subspecialty	Disease	Task	Sub-task	Complexity	Level of study
Physiology	Cardiovascular	SLE	Diagnosis	Hemodynamic	Three-jump	Senior level

Question Stem

A **40-year-old woman** with long-standing SLE presents with **dyspnea and chest pain**. Echocardiography shows **pericardial effusion without tamponade physiology**.

Which of the following physiological changes would be expected if the effusion progressed to **cardiac tamponade**?

Options

A. Reduced venous return and decreased stroke volume
B. Increased preload with enhanced cardiac output
C. Elevated systolic pressure with widened pulse pressure
D. Decreased right atrial pressure with increased ventricular filling
E. Enhanced ventricular compliance improving diastolic filling

Correct answer: A. Reduced venous return and decreased stroke volume

Explanation:

Correct (A): Tamponade restricts ventricular filling due to pericardial pressure → decreased venous return and reduced stroke volume → hypotension.

B. Increased preload: Opposite of tamponade physiology.

C. Elevated systolic pressure: Tamponade causes low, not high, systolic pressure; pulse pressure narrows.

D. Decreased RA pressure: RA pressure is increased, not decreased, in tamponade.

E. Enhanced compliance: Ventricular compliance decreases, not increases.
